# Effect of AZD4017, a Selective 11β-HSD1 Inhibitor, on Bone Turnover Markers in Postmenopausal Osteopenia

**DOI:** 10.1210/clinem/dgac100

**Published:** 2022-03-11

**Authors:** Afroze Abbas, Marian Schini, Gemma Ainsworth, Sarah R Brown, Jamie Oughton, Rachel K Crowley, Mark S Cooper, Rebecca J Fairclough, Richard Eastell, Paul M Stewart

**Affiliations:** Faculty of Medicine and Health, University of Leeds, and Leeds Teaching Hospitals NHS Trust, Leeds LS9 7TF, UK; Academic Unit of Bone Metabolism, University of Sheffield, Sheffield S5 7AU, UK; Clinical Trials Research Unit, University of Leeds, Leeds LS2 9JT, UK; Clinical Trials Research Unit, University of Leeds, Leeds LS2 9JT, UK; Clinical Trials Research Unit, University of Leeds, Leeds LS2 9JT, UK; School of Medicine and Medical Science, University College Dublin, Dublin, Ireland; Concord Clinical School, Faculty of Medicine & Health, University of Sydney, NSW 2139, Australia; Emerging Innovations Unit, Discovery Sciences, BioPharmaceuticals R&D, AstraZeneca, Cambridge, UK; Academic Unit of Bone Metabolism, University of Sheffield, Sheffield S5 7AU, UK; Faculty of Medicine and Health, University of Leeds, and Leeds Teaching Hospitals NHS Trust, Leeds LS9 7TF, UK

**Keywords:** osteopenia, osteocalcin, 11β-HSD1, glucocorticoids, AZD4017

## Abstract

**Context:**

The causative link between circulating glucocorticoid excess and osteoporosis is well-established. The enzyme 11β-hydroxysteroid dehydrogenase type 1 (11β-HSD1), which increases local cortisol production, is expressed in human osteoblasts and its activity increases with age.

**Objective:**

We hypothesized that local 11β-HSD1 might mediate an age-related decrease in bone formation and that selective 11β-HSD1 inhibition may enhance bone formation.

**Methods:**

A dual-center, phase II, randomized, double-blind, placebo-controlled trial of 90 days’ treatment with AZD4017 (a selective 11β-HSD1 inhibitor) was conducted in 55 postmenopausal women with osteopenia. Participants received 400 mg oral AZD4017 twice daily vs matched placebo over 90 days. The primary outcome measure was the impact on the bone formation marker osteocalcin. Secondary objectives included correlation with 11β-HSD1 activity.

**Results:**

At 90 days, osteocalcin levels did not differ between treatment groups: active (mean 22.3 [SD 8.6] ng/mL, n = 22) and placebo (21.7 [SD 9.2] ng/mL, n = 24), with a baseline-adjusted treatment effect of 0.95 (95% CI: −2.69, 4.60). The results from the urinary [THF + alloTHF]/THE ratio (index of 11β-HSD1 activity) and the urinary cortisol/cortisone ratio (index of 11β-HSD2 activity) confirmed a > 90% inhibition of 11β-HSD1 but no change in activity of 11β-HSD2.

**Conclusion:**

This trial demonstrates that AZD4017 selectively inhibits 11β-HSD1 activity in vivo in a safe and reversible manner. Following 90 days of treatment, there is no effect on bone formation, indicating that the relative impairment of bone mineral density in postmenopausal women is not mediated by local intracellular production of cortisol under normal physiological concentrations.

Age-related osteoporosis is a major public health problem. It is estimated that approximately 1 in 3 women and 1 in 5 men older than 50 years will have a fracture during their remaining lifespans. In 2017, the annual fracture-related costs in Europe were estimated to be €37.5 billion, a figure that is expected to increase by 27% by 2030. The disability-adjusted life years (DALYs) per 1000 individuals older than 50 years were estimated at 21 years; this figure is higher than analogous estimates for diseases such as stroke or chronic obstructive pulmonary disease (1). Severe osteoporosis is a well-recognized complication of therapeutic or endogenous glucocorticoid excess. Oral glucocorticoids increased the risk of a vertebral fracture by almost 3-fold (relative rate [RR] 2.86; 95% CI, 2.56–3.16) in one study, while doubling the risk of hip fracture (RR 2.01; 95% CI, 1.74–2.29) (2).

Glucocorticoids exert their adverse effects on bone through a range of mechanisms, but the main adverse impact appears to be a decrease in bone formation through a direct effect on osteoblasts. By interfering with several important signaling pathways, glucocorticoids cause the differentiation of precursor cells into adipocytes, ultimately decreasing the number of osteoblasts (3). Glucocorticoids also cause increased production of inhibitors of Wnt signaling, such as sclerostin, leading to a reduction in bone formation (4, 5). Moreover, high doses of corticosteroids can lead to osteoblast apoptosis (6). Glucocorticoids transiently cause enhanced bone resorption through increased expression of the receptor activator of nuclear factor-κΒ ligand (RANKL) on osteoblasts. The increase in the RANKL to osteoprotegerin ratio can affect the differentiation of osteoclasts (4). These processes lead to an early phase of rapid bone loss due to the RANKL stimulation, then to a more sustained uncoupling of bone formation due to a decrease in osteoblastogenesis and an increase of apoptosis. Other endocrine factors that can contribute to steroid-induced osteoporosis include suppression of insulin-like growth factor 1 (IGF1), and collagen degradation (7). Growth hormone (GH), a potent anabolic hormone, can be decreased as an effect of glucocorticoids (8), as can the production of gonadotropins (9). Finally, glucocorticoids can adversely affect calcium metabolism and muscle function, leading to sarcopenia with an increased likelihood of falls (3).

Over and above circulating cortisol concentrations, local tissue metabolism of glucocorticoids, mediated through the activity of a pair of 11β-hydroxysteroid dehydrogenase (11β-HSD) enzymes, represents an important pre-receptor regulator of corticosteroid hormone action. These enzymes interconvert hormonally inactive cortisone with active cortisol. The 11β-HSD1 enzyme is intrinsically bidirectional but primarily acts in vivo as a reductase, converting cortisone to cortisol. This enzyme has a widespread localization in tissues, including liver and adipose tissue, and its activity has been linked to diseases such as hepatic steatosis, diabetes mellitus, and central obesity. In contrast, the 11β-HSD2 enzyme is unidirectional, inactivating cortisol to cortisone. It is localized in aldosterone-sensitive tissues, such as the kidney, where it protects the mineralocorticoid receptor (MR) from cortisol excess, thus conferring specificity for aldosterone on the MR (10, 11).

Human bone also expresses 11β-HSD1 (but not 11β-HSD2) primarily within the osteoblasts (12). Osteoblasts grown ex vivo are capable of generating active glucocorticoids, with 11β-HSD1 expression and activity increasing dramatically with age (13). Osteocalcin, a bone turnover marker secreted by mature osteoblasts, was found to be negatively correlated with serum cortisone but not serum cortisol. Individuals aged 61-73 years with low cortisone levels (and thus lower 11β-HSD1 activity) had higher levels of osteocalcin; a 9.4 nmol/L increase in cortisone (1 SD) was associated with a 6.7% decrease in osteocalcin (14). The use of a nonselective 11β-HSD1 inhibitor (carbenoxolone) in a small (n = 8) 7-day study, showed a decrease of bone resorption markers of about 10% (12).

AZD4017 is a novel orally bioavailable inhibitor of 11β-HSD1 enzyme activity. It is selective (> 2000×) for 11β-HSD1 over human recombinant 11β-HSD2 and other closely- homologous enzymes in vitro. Reports have confirmed its inhibitory potency on 11β-HSD1 when used in clinical studies for other indications (15-17).

We hypothesized that 11β-HSD1 activity is a physiological regulator of bone remodeling imbalance and that its therapeutic inhibition could reverse the changes seen with age. The primary aim of this study was to examine the effect of AZD4017 on the levels of biochemical markers of bone formation in postmenopausal women with osteopenia.

## Methods

### Design

This was a double-blind placebo-controlled randomized (1:1) clinical trial to assess the efficacy, safety, and tolerability of AZD4017 400 mg (2 × 200 mg tablets) twice daily (morning and evening) administered for 90 days in participants with postmenopausal osteopenia. The control group received matching placebo tablets taken orally twice daily (morning and evening). The study was performed in 2 centers: the National Institute for Health Research Musculoskeletal Biomedical Research Centre (NIHR-BRC) in Leeds and the National Institute for Health Research Clinical Research Facility (NIHR-CRF) in Sheffield, both in the UK.

### Ethics

The trial was conducted in accordance with the principles of Good Clinical Practice (GCP) in clinical trials, as applicable under UK regulations, the NHS Research Governance Framework (RGF) and through adherence to CTRU Standard Operating Procedures (SOPs). The study was approved by South Central—Oxford B Research Ethics Committee (13/SC/0523), institutional review boards of participating centers, and the Medicines and Healthcare Products Regulatory Agency. The trial is registered on the ISRCTN trials database (ISRCTN32813419) and EudraCT database (EudraCT 2013-003387-32).

Following provision of written informed consent, participants attended the units for a screening visit to exclude secondary causes of bone loss and vitamin D deficiency. This visit included recording of demographic data, medical history, medications taken currently and within last 2 years, medication allergy history, date of last menses, and recreational drug use history. Clinical measurements were performed that included: blood pressure, heart rate, height, weight, body mass index (BMI) calculation, and electrocardiogram (ECG). If any participant reported recent height loss or back pain suggestive of vertebral fracture, they underwent physical examination. Bone mineral density measurements (BMD) of the hip and lumbar spine were also performed. A scan of the distal forearm was performed if one of the other sites was unsuitable. Blood samples were taken to assess eligibility. Any participant found to have a serum 25-hydroxyvitamin D [25(OH)D] level lower than 20 nmol/L was excluded from the study and the results were forwarded to their general practitioner. If the 25(OH)D level was between 20 and 50 nmol/L, the patient was asked to return for a further visit (visit VD1) and was given a dose of 100 000 IU cholecalciferol (vitamin D_3_) orally. They were then asked to return for visit VD2, 14 to 21 days later, for a repeat blood sampling. If the result remained less than 50 nmol/L, a second loading dose was given at visit VD3. Another blood sampling after this second dose was then performed at visit VD4, 14 to 21 days later. No other procedures were carried out at these additional visits. If a participant had not achieved a 25(OH)D level greater than 50 nmol/L after 2 loading doses of vitamin D_3_, they were excluded from further participation.

A screening period of a minimum of 7 days and a maximum of 30 days followed, prior to the baseline visit (randomization). Where vitamin D replacement was necessary, this screening period could be extended to 60 days. The participants were then randomly assigned to receive either AZD4017 or placebo in a 1:1 ratio. Randomization was performed centrally through the University of Leeds Clinical Trials Research Unit (CTRU). To ensure that equal numbers of participants were randomized to the 2 treatment arms, a random permuted blocks design was used. In order to maintain blind status, the statistician was unaware of the treatment allocation until all participants had completed the 90-day treatment period. Participants were asked to keep a record of the time of drug dose using a diary of events, any doses missed, and to bring empty bottles and any leftover study medication with them to the next visit.

Randomized participants then entered a 90-day double-blind treatment period with visits at 7(±1), 28(±4), 56(±4), and 90(±6) days. A follow-up visit at 90 ± 14 days after the last dose of study medication was required for all participants for completion of the study.

Patients were discontinued from the study medication if they had a significant or serious adverse event at the discretion of the principal investigator, if they experienced hepatotoxicity as defined by alanine aminotransferase (ALT) and/or aspartate transaminase (AST) > 3× upper limit of normal (ULN) or bilirubin > 3× ULN for 2 consecutive samples or isolated sample of 5× ULN ALT or AST, and/or muscle toxicity as defined by creatine kinase (CK) > 5× ULN or myopathy (associated muscle symptoms with no other explanatory cause), or rhabdomyolysis (myopathy with associated evidence of renal damage).

### Study Participants

The study included postmenopausal women (> 50 years of age and amenorrhea for > 12 months) with osteopenia, defined as a BMD T-score at the lumbar spine or total hip (whichever had the lower value) below or equal to −1 and greater than −2.5 for which placebo treatment would not be considered detrimental. Exclusion criteria were: clinical or biochemical evidence of secondary osteoporosis; current or recent use (within the last 2 years) of medication likely to have an impact on bone (estrogens, aromatase inhibitors, bisphosphonates, fluoride, denosumab, anabolic agents, strontium ranelate, anticonvulsants, or oral, inhaled, or nasal glucocorticoids); bilateral fractures of the radius and/or tibia; women of child-bearing potential; use of recreational drugs; uncontrolled hypertension; a diagnosis of any inflammatory disorder that could require treatment with glucocorticoids during the course of the study; gastrointestinal, hepatic, or renal disease or any other condition known to interfere with absorption, distribution, metabolism, or excretion of drugs; medical/surgical procedure or trauma within 4 weeks; known hypersensitivity or intolerance to 11β-HSD1 inhibitors or to AZD4017; blood measurement abnormalities (CK more than 2 times the ULN on 2 consecutive measurements, ALT and/or AST > 1.5 ULN, alkaline phosphatase [ALP] > ULN, bilirubin (total) >1.5 ULN). Participants already on calcium/vitamin D supplementation were asked to continue this for the duration of the study, as alterations in calcium administration could impact measured change in osteocalcin, which was the primary endpoint.

Potential trial participants were identified from those who had previously attended for dual-energy x-ray absorptiometry (DXA) scans at Leeds Teaching Hospitals NHS Trust. The Sheffield participants were identified from a cohort who previously agreed to participate in osteoporosis trials but were found to have osteopenia when screened.

### Endpoints

The primary endpoint was the change from baseline in the bone formation marker serum osteocalcin (OC) following 90 days of treatment with AZD4017 or placebo.

The main secondary endpoint for the study was the change from baseline in bone resorption marker serum C-terminal cross-linked telopeptide (βCTX) following 90 days of treatment with AZD4017 or placebo. Further secondary endpoints included: 1) the evaluation of the ratio of the principal urinary metabolites of cortisol (tetrahydrocortisol [THF] and allo-tetrahydrocortisol [alloTHF]) to those of cortisone (tetrahydrocortisone [THE]), that is, the (THF + alloTHF)/THE ratio, (a) to assess 11β-HSD1 activity in order to examine the potency of AZD4017, and (b) to determine whether changes in biochemical markers of bone turnover correlated with baseline or the change in systemic 11β-HSD1 activity; and 2) safety and tolerability of AZD4017 as assessed by blood tests (urea and electrolytes, liver function tests, creatine kinase, thyroid function tests) measurements and documentation of adverse events. This included an analysis of treatment compliance.

The exploratory outcome variables were: BMD measurements by DXA; bone marker levels at each study time point, including osteocalcin and βCTX as described above, bone alkaline phosphatase (BAP), and procollagen type 1 N-terminal propeptide (PINP); and change in grip strength (determined with a handheld dynamometer), a 6-meter walk test, and the time taken to complete 5 chair stands between baseline and 90 days to examine the change in muscle strength.

Any ongoing adverse events at the follow-up visit were followed until resolution, until the adverse event stabilized, until it was otherwise explained, or until the participant was lost to follow-up.

### Laboratory Measurements

The maximum volume of blood required from the study participants at each visit was 30 mL. For those individuals who required vitamin D dosing prior to receiving AZD4017 treatment, an additional 5 mL of blood was taken at VD2 and then VD4 (if required).

Biomarker samples were examined at the Sheffield site. CTX (coefficient of variation [CV] 5.2%), PINP (CV 4.8%), and osteocalcin (CV 1.6%) were measured using the Cobas e411 analyzer (Roche Diagnostics) and bone ALP (CV 5.5%) was measured using the iSYS analyzer (Immunodiagnostic Systems).

The 24-hour urine volume was recorded, and aliquots stored at −20 °C until analysis using gas chromatography/ mass spectrometry (GC/MS). Urinary free cortisol/free cortisone ratio (UFF/UFE) was used as a measure of 11β-HSD2 activity as previously validated by our group (18). If unchanged, the ratio of (THF + alloTHF)/THE was then used as an accurate measure of 11β-HSD1 activity (18). As a surrogate measure of 24-hour cortisol secretion rate, total urinary cortisol metabolites were presented as a summation of UFF, UFE, THF, alloTHF, THE.

### Imaging

Bone mineral density (BMD) measurements at the spine and hip were performed at baseline, 90 days, and 180 days. A scan of the distal forearm was performed if one of the other sites was unsuitable. The scans were performed in Hologic Discovery A (Sheffield) and Lunar iDXA (Leeds). All scanning was performed in accordance with standard operating procedures.

### Additional Tests

Additional tests to investigate impact on physical performance were undertaken. These were the repeated chair stand test (in which the participant was asked to repeatedly stand from a seated position and then sit down, as quickly as possible 5 times), a 6-meter walk test (participants were asked to walk to their usual pace from a start point to the other end of the course and back again), and a hand grip strength test using a hand dynamometer.

### Sample Size and Statistical Analysis

The sample size for the primary endpoint was based on previous studies examining the relationship between serum osteocalcin and cortisol levels in elderly participants in which the mean (SD) level of osteocalcin was 8.5 μg/L (1.4 μg/L) (14). A sample size of 43 participants in each arm would enable a difference of 10% in osteocalcin to be detected between groups at 90 days with 80% power (type II error β = 0.2) using a 2-sample *t* test at the 5% significance level (α = 0.05). An interim analysis was planned after 40 participants. Accounting for the interim analysis and allowing for 10% dropout, a total sample size of 100 participants was required (with significance level α = 0.0051 for the interim analysis and α = 0.0481 for the final analysis respectively).

The primary analysis of efficacy followed a modified intention-to-treat (mITT) population based on data from all randomized participants who took at least one dose of study medication and contributed sufficient data for at least one efficacy endpoint to be calculated. All participants who received at least one dose of study medication, and for whom any postdose data are available, were included in the safety population.

Individual linear regression analyses were conducted for serum osteocalcin at each time point, adjusting for baseline serum osteocalcin. Descriptive summary statistics were also produced for each time point. Similar analyses were performed for the secondary endpoints.

The relationship between 11β-HSD1 at visit 5 (90 days) and baseline 11β-HSD1 or its change as inferred by change in the urinary [THF + alloTHF]/THE ratio was analyzed using linear regression analysis. Post hoc nonparametric Wilcoxon rank sum tests were also implemented.

## Results

**Figure 1.**
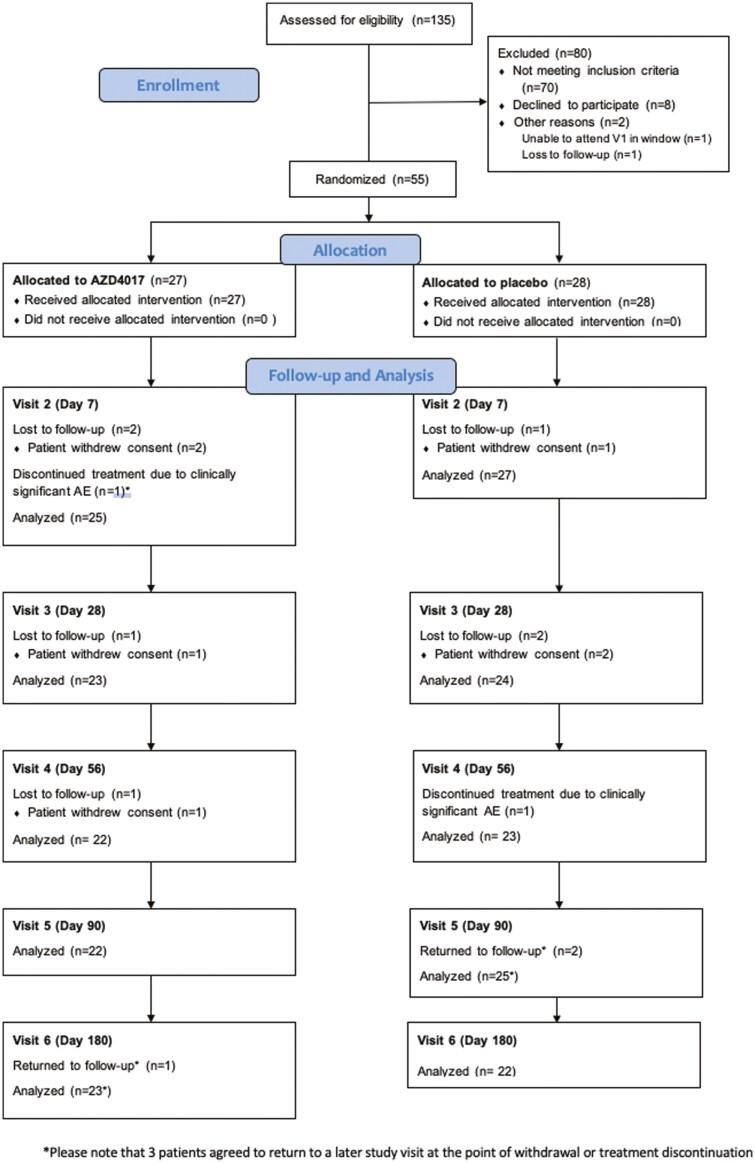
Consort diagram of the study showing recruitment and study flow.

Between May 28, 2015, and March 31, 2018, 135 patients were assessed for eligibility across 2 different UK sites (Chapel Allerton Hospital, Leeds and Northern General Hospital, Sheffield). Fifty-five (40.7%) of these patients were randomized, 27 to receive AZD4017 and 28 to receive placebo (Fig. 1). Of these, 11 patients withdrew from the study during the follow-up period, although 1 of these participants withdrew after providing the data required for analysis of the primary endpoint.

The original target sample size of 100 patients was not reached due to a combination of slow recruitment and a higher-than-expected noneligible rate. The preplanned interim analysis was amended to incorporate a futility assessment and was performed after data were available on 52 patients. The interim analysis indicated futility for the primary endpoint, therefore the Data Monitoring and Ethics Committee (DMEC) recommended that the trial should close to recruitment, having recruited a total of 55 participants. The DMEC was composed of experienced academics and clinicians who were independent of the study teams. No formal statistical testing to include *P* values was conducted at final analysis due to the small sample size.

Baseline characteristics are shown in Table 1.

**Table 1.** Baseline characteristics of the study population

	Active treatment AZD4017 (n = 27)	Placebo (n = 28)
**Age, years**	64.1 (7.4)	65.8 (9.3)
**BMI, kg/m** ^**2**^	26.6 (4.0)	28.1 (4.5)
**Systolic blood pressure, mmHg**	130.2 (12.5)	135.9 (14.8)
**Diastolic blood pressure, mmHg**	73.9 (9.8)	76.2 (8.2)
**Baseline T-score, lumbar spine**	−1.8 (0.4)	−1.6 (0.6)
**Baseline T-score, total hip**	−1.2 (0.6)	−1.0 (0.6)
**Creatinine, µmol/L**	64.1 (7.7)	67.1 (11.2)
**Urea, mmol/L**	5.7 (1.4)	5.4 (1.4)
**Sodium, mmol/L**	140.8 (2.1)	141.0 (2.1)
**Vitamin D, nmol/L**	75.3 (26.0)	72.2 (22.0)
**TSH, mIU/L**	1.9 (1.3)	2.0 (1.2)
**Free T4, pmol/L**	14.1 (2.1)	14.6 (1.9)
**Bilirubin, µmol/L**	9.2 (3.8)	10.1 (5.1)
**ALT, IU/L**	18.2 (3.9)	18.6 (5.7)
**Albumin, g/L**	42.5 (3.4)	42.7 (3.9)
**ALP, IU/L**	93.0 (46.4)	90.7 (36.0)
**GGT, IU/L**	25.0 (22.3)	24.6 (16.1)
**LDH, IU/L**	414.4 (69.9)	426.7 (78.9)
**CK, IU/L**	105.1 (43.9)	103.7 (59.2)

Data are presented as mean (SD).

Abbreviations: ALT, alanine aminotransferase; AST, aspartate aminotransferase; BMI, body mass index; CK, creatine kinase; GGT, gamma-glutamyl transferase; LDH, lactate dehydrogenase; T4, thyroxine; TSH, thyrotropin (thyroid stimulating hormone).

### Primary Endpoint (Osteocalcin)

**Figure 2.**
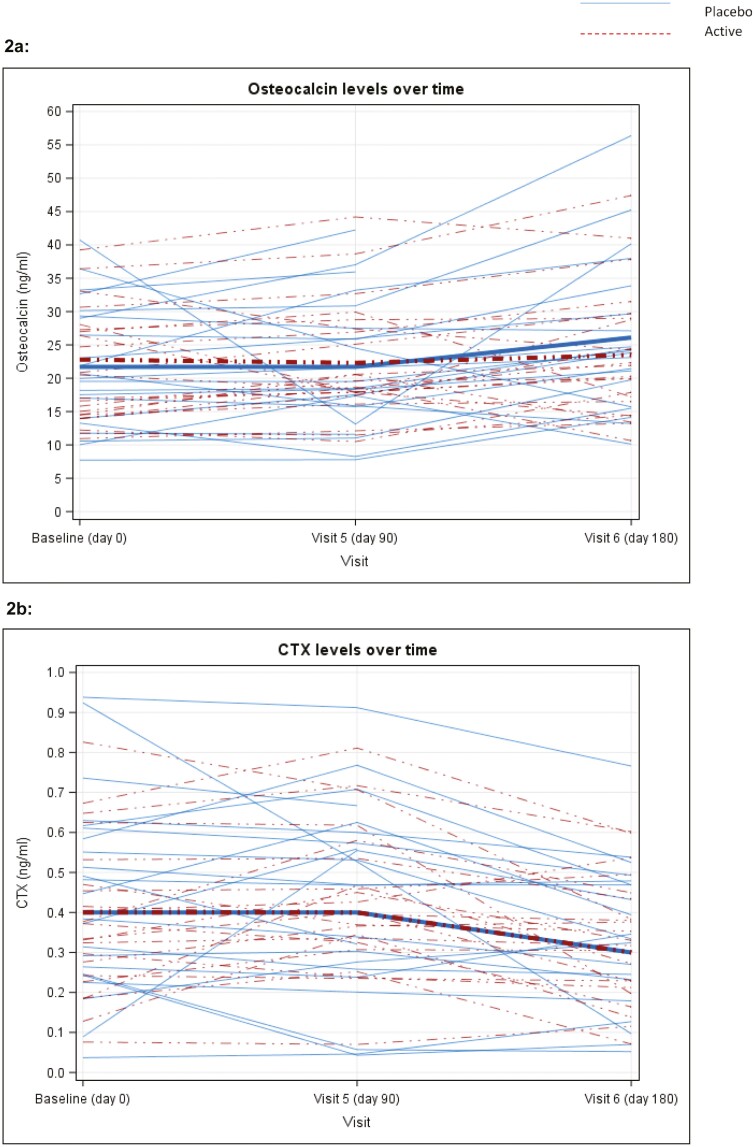
Plot of osteocalcin (Fig. 2a) and CTX (Fig. 2b) for each patient by study visit and treatment group. Mean values for each group represented by thick lines, with numerical data shown in Table 2.

Figure 2A displays individual patient osteocalcin levels at baseline, 90, and 180 days, with values given in Table 2. At 90 days absolute osteocalcin measurements were similar between active (mean 22.3 [SD 8.6] ng/mL, n = 22) and placebo (21.7 [SD 9.2] ng/mL, n = 24) groups, with an estimated adjusted-for-baseline treatment effect of 0.95 [95% CI: −2.69, 4.60] from linear regression analysis, for patients taking AZD4017.

**Table 2.** Bone density and bone turnover markers at baseline, day 90 and day 180 for active group (AZD4017) vs placebo group

	Baseline		Day 90		Day 180	
	Active	Placebo	Active	Placebo	Active	Placebo
**T-score lumbar spine**	−1.8 (0.4) n = 26	−1.6 (0.6) n = 27	−1.8 (0.4) n = 21	−1.8 (0.6) n = 24	−1.8 (0.4) n = 21	−1.8 (0.5) n = 20
**T-score total hip**	−1.2 (0.6) n = 27	−1.0 (0.6) n = 28	−1.2 (0.8) n = 22	−0.9 (0.8) n = 23	−1.1 (0.7) n = 23	−1.0 (0.7) n = 22
**Serum osteocalcin (ng/mL)**	22.8 (9.5) n = 27	21.7 (8.8) n = 27	22.3 (8.6) n = 22	21.7 (9.2) n = 24	23.5 (9.4) n = 23	26.1 (11.1) n = 22
**Serum βCTx (ng/mL)**	0.4 (0.2) n = 27	0.4 (0.2) n = 27	0.4 (0.2) n = 22	0.4 (0.2) n = 24	0.3 (0.2) n = 23	0.3 (0.2) n = 22
**Serum BAP (ng/mL)**	19.0 (5.2) n = 27	19.8 (6.3) n = 27	17.7 (4.6) n = 21	20.1 (5.6) n = 24	18.1 (4.1) n = 23	18.1 (5.4) n = 22
**Serum P1NP (ng/mL)**	48.2 (18.0) n = 27	47.0 (16.2) n = 27	51.4 (19.1) n = 22	48.6 (19.8) n = 24	46.7 (15.0) n = 23	50.5 (21.7) n = 22

Data are presented as mean (SD).

Abbreviations: BAP, bone alkaline phosphate; CTX, C-terminal cross-linked telopeptide; PINP, procollagen I intact N-terminal propeptide.

### Secondary Endpoints

Figure 2B displays individual patient CTX levels at baseline, 90, and 180 days with numerical values at baseline, day 90, and day 180 given in Table 2.

At 90 days CTX levels were similar between the active (mean 0.4 [SD 0.2] ng/mL, n = 22) and placebo (0.4 [SD 0.2] ng/mL, n = 24) groups, with an estimated baseline-adjusted treatment effect of 0.04 (95% CI: −0.04, 0.12).

**Figure 3.**
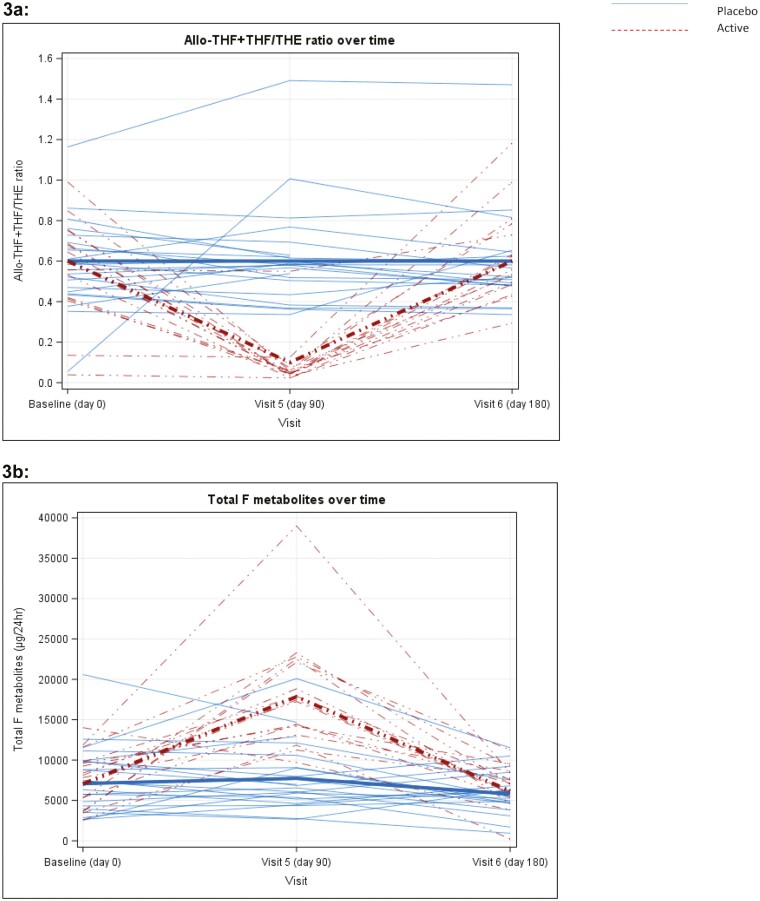
3a, Plot of urinary (THF + alloTHF)/THE for each patient by study visit and treatment group (baseline [n = 49], day 90 [n = 42], day 180 [n = 43]). Mean values for each group represented by thick lines. 3b, Plot of total urinary cortisol (F) metabolites for each patient by study visit and treatment group. Mean values for each group represented by thick lines.

In the placebo group, the urinary (THF + alloTHF)/THE ratio remained similar throughout the study; however, in the AZD4017-treated group the ratio decreased dramatically at 90 days (mean 0.1 [SD 0.1], n = 19) vs placebo (mean 0.6 [SD 0.3], n = 23), (Fig. 3A), with a 90-day baseline-adjusted treatment effect of −2.31 (95% CI: −2.69, −1.92). These changes were reversible after the treatment ended. Levels were similar across both arms at each time point, with an estimated baseline-adjusted 90-day treatment effect of −0.05 (95% CI: −0.20, 0.11).

Urinary free cortisol/free cortisone ratio (UFF/UFE ratio) was used as a surrogate marker of 11β-HSD2 activity. At 90 days, this was similar between the active (mean 0.6 [SD 0.3], n = 19) vs placebo (mean 0.6 [SD 0.2], n = 23). Taken together this confirmed that that AZD4017 successfully inhibited 11β-HSD1 in a selective manner and did not affect 11β-HSD2 activity.

Analysis of the urinary alloTHF/THF ratio, a marker of the 5α/5β reductase activity, showed a trend for a small reduction at 90 days in patients receiving AZD4017, with an estimated adjusted-for-baseline treatment effect of −0.19 (95% CI: −0.34, −0.04). Results should, however, be interpreted with caution, as the model demonstrated poor fit.

As anticipated following selective 11β-HSD1 inhibition and increased cortisol clearance, there was a concomitant increase in cortisol secretion rate to maintain normal circulating cortisol concentrations (Fig. 3B). This was demonstrated through an increase in total urinary cortisol metabolites (μg/24 h) on day 90 following AZD4017 treatment (17 856 [SD 6674] vs 7736 [SD 4091] placebo, and vs 7069 [SD 3008] baseline) that again reverted to normal 90 days after cessation.

Linear regression analysis of serum osteocalcin at 90 days adjusting for the change from baseline in 11β-HSD1 activity at 90 days, indicated that the degree of 11β-HSD1 inhibition was a poor predictor of osteocalcin.

### Other Exploratory Endpoints

Other exploratory bone parameters remained stable and did not differ between groups. This included BMD at both the spine and total hip, BAP, and PINP (see Table 2). There was also no difference between the groups for grip strength, 6-meter walk, and chair stand test results (data not shown).

### Safety and Adverse Events

AZD4017 was well tolerated with no serious adverse reactions (SARs) or suspected unexpected adverse reactions (SUSARs). There were 5 serious adverse events in the trial, 1 in the AZD4017 arm (prolapsed disc) and 4 in the placebo arm (left broken ankle, laceration to right knee, popliteal deep vein thrombosis, and right lacunar stroke), none of which were thought to be related to trial treatment. Milder adverse effects were similar across the AZD4017 and placebo groups. Safety parameters, including biochemical tests of renal and hepatic function, heart rate, and systolic and diastolic blood pressure, showed no changes.

## Discussion

The ability to arrest and/or reverse postmenopausal osteopenia would be a major health advance. The clinical and current unmet need is real and becoming more prevalent in aged population.

Based on exciting preclinical data and a sound hypothesis, this academic-industry partnership has evaluated inhibition of 11β-HSD1 as a candidate pathway, in a phase II placebo-controlled trial. Building on published data, AZD4017 was shown to be safe, highly effective, and selective (in that it had no effect on 11β-HSD2 activity), inhibiting 11β-HSD1 by > 90% (19, 20). Despite recruitment issues and early trial cessation, the data demonstrated no significant effect of a 90-day course of treatment with AZD4017 on any bone marker, and specifically no change in the glucocorticoid-responsive marker of bone formation, serum osteocalcin.

The rationale for exploring 11β-HSD1 as a target was based on clinical observations of patients with both endogenous and exogenous Cushing syndrome, in whom osteopenia and osteoporosis leading to fracture are common clinical findings, together with our work showing age-related increase in expression of 11β-HSD1 in osteoblasts (21). Cortisol excess is catabolic at sites other than bone; in skin and muscle, for example, we have demonstrated significant improvements in age-related skin changes, improved wound healing, and protection against muscle wasting with 11β-HSD1 inhibition (22, 23). Other clinically based studies have shown that some of the metabolic conditions associated with glucocorticoid excess, for example diabetes mellitus and hyperlipidemia, are also improved following 11β-HSD1 inhibition (24).

A key factor here is likely to be exposure from circulating excess vs autocrine generation of active cortisol at a tissue level. While the data linking cortisol/glucocorticoid excess to poor bone health is irrefutable, it is based on prolonged and inappropriate exposure to excessive circulating levels of glucocorticoid. The relationship between bone markers and bone mineral density and “physiological” glucocorticoid levels is less clear. There is little evidence for excess cortisol secretion in osteoporosis. For example, in a study of women with vertebral fractures there was no increase in urinary total or free cortisol (25) and clinical guidelines do not support routine screening for Cushing syndrome in patients with osteoporosis (26). A key attraction of selective 11β-HSD1 inhibition is that changes to systemic cortisol metabolism are offset by concomitant increases in secretion rate; circulating concentrations are unaltered. The data from our study endorse the suggestion that across physiological conditions, local changes to cortisol concentrations mediated by 11β-HSD1 expression in osteoblasts are not rate limiting in regulating bone formation (or resorption). Under conditions of glucocorticoid excess, a different picture might pertain; 11β-HSD1 is induced by glucocorticoids themselves in a fast-forward mechanism and 11β-HSD1 inhibition might “protect” the skeleton from the added deleterious effects of local cortisol production in endogenous Cushing syndrome. Furthermore, because the 11β-HSD1 kinetics for metabolizing inactive prednisone to active prednisolone are identical to those of cortisone-cortisol, it is interesting to speculate that this might also offer a steroid-sparing bone therapy in patients treated with exogenous steroids. There are preliminary phase I data supporting such a concept, AZD4017 blocking the prednisolone-induced suppression of serum osteocalcin in healthy males (27).

Additional factors need to be considered in explaining lack of efficacy. We have only assessed 11β-HSD1 inhibition systemically through the measurement of 24-hour urinary steroid profiles that undoubtedly are heavily influenced by hepatic and renal metabolism. But in other settings where similar analyses have been endorsed by tissue 11β-HSD1 activity studies (eg, adipose tissue, skin), enzyme inhibition has been seen and there are no reasons why osteoblasts would not be exposed to therapeutic inhibitor concentrations. Similarly, the effects of glucocorticoids on osteocalcin are rapid and persist over exposure. When prednisolone is given to healthy subjects, the nadir in osteocalcin is reached within 4 days, with osteocalcin levels returning to baseline levels within 3 days upon cessation (28); our repeated analysis over a 90-day period would suffice to detect any differences.

In postmenopausal women with osteopenia and with an intact and normally functioning hypothalamic-pituitary-adrenal axis, selective 11β-HSD1 inhibitors have no demonstrable or clinically significant effect on bone formation. A change in local glucocorticoids in the face of normal circulating cortisol concentrations is unlikely to be rate limiting in altering bone metabolism. Further bone-sparing studies are now indicated to determine if selective 11β-HSD1 inhibitors might arrest or reverse the effects of cortisol excess in endogenous Cushing syndrome or in patients treated with prednisolone.

## Data Availability

Restrictions apply to the availability of data generated or analyzed during this study to preserve the confidentiality of the participants. The corresponding author will, on request, detail the conditions under which access to data may be provided.

## Abbreviations

11β-HSD: 11β-hydroxysteroid dehydrogenase
25(OH)D: 25-hydroxyvitamin D
alloTHF: allotetrahydrocortisol
ALT: alanine aminotransferase
AST: aspartate aminotransferase
BAP: bone alkaline phosphate
BMD: bone mineral density
CK: creatine kinase
CTX: C-terminal cross-linked telopeptide
CV: coefficient of variation
DXA: dual-energy x-ray absorptiometry
MR: mineralocorticoid receptor
PINP: procollagen I intact N-terminal propeptide
RANKL: receptor activator of nuclear factor-κB ligand
RR: relative rate
THE: tetrahydrocortisone
THF: tetrahydrocortisol
ULN: upper limit of normal
VD#: visit day #

## Clinical Trial Information

This trial is registered in the ISRCTN trials database (ISRCTN32813419) and EudraCT database (EudraCT 2013-003387-32).
